# Supplementary material for: Integrative Multi-Omics Analysis of the Rumen in Tan Sheep with Contrasting Average Daily Gain
Source: Microorganisms. 2025 Dec 18;13(12):2882. doi: 10.3390/microorganisms13122882 (PMC12736088; doi:10.3390/microorganisms13122882)
Supplement: Supplementary file 1 [file microorganisms-13-02882-s001.zip › microorganisms-4020132-supplementary.pdf]

# Analysis and Correlation of the Growth Performance, Blood biochemical features, Rumen metabolomics, and Rumen microbiome differences between High and Low ADG Tan sheep

**Hao Zheng [1], Xiaohong Han [1], Wenjuan Shen [1], Xinrui Zhang [1], An Shi [1], Tonggao Liu [2], Chong Yang[2] and Jinzhong Tao [1]***

[1] College of Animal Science and Technology, Ningxia University, Yinchuan 750021, China; 13213619969@163.com(H.Z.);hanxh1254@126.com(H.H.); shenwenjuan0518@163.com(W.S.);iszhangxinr@163.com(X.Z.);shian_1988@outlook.com(A.S.);tao_jz@nxu.edu.cn(J.T.)

[2] Animal Husbandry Workstation of Ningxia, Yinchuan 750021, China; 541876150@qq.com(T.L.);xmjych7203@163.com(C.Y.)

* Correspondence: tao_jz@nxu.edu.cn; +86 13639571792

## Supplementary material

**Table S1**.Analysis of differences in visceral organ weights between high and low ADG Tan sheep

| Item | HADG | LADG | P-value |
|---|---|---|---|
| Testicle weight/kg | 0.22±0.05 | 0.22±0.05 | 0.925 |
| Proportion of testicle weight to live weight before slaughter/% | 0.45±0.07 | 0.52±0.11 | 0.252 |
| Heart weight/kg | 0.24±0.05 | 0.2±0.03 | 0.227 |
| Proportion of heart weight to live weight before slaughter/% | 0.48±0.08 | 0.48±0.04 | 0.996 |
| Liver weight/kg | 0.89±0.04 | 0.64±0.34 | 0.155 |
| Proportion of liver weight to live weight before slaughter/% | 1.8±0.1 | 1.51±0.79 | 0.431 |
| Spleen weight/kg | 0.05±0.01 | 0.05±0.01 | 0.771 |
| Proportion of spleen weight to live weight before slaughter/% | 0.1±0.02 | 0.11±0.03 | 0.464 |
| Lung weight/kg | 0.56±0.11 | 0.46±0.07 | 0.111 |
| Proportion of lung weight to live weight before slaughter/% | 1.14±0.16 | 1.09±0.12 | 0.627 |
| Rumen fat weight/kg | 0.5±0.27 | 0.36±0.25 | 0.419 |
| Proportion of rumen fat weight to live weight before slaughter/% | 0.98±0.48 | 0.84±0.53 | 0.678 |
| Kidney weight/kg | 0.13±0.01A | 0.11±0.01B | 0.002 |
| Proportion of kidney weight to live weight before slaughter/% | 0.27±0.01 | 0.26±0.02 | 0.251 |
| Perirenal fat weight/kg | 0.15±0.05 | 0.12±0.05 | 0.342 |

| | | | |
|---|---|---|---|
| Proportion of perirenal fat to live weight before slaughter/% | 0.3±0.08 | 0.27±0.1 | 0.712 |
